# Anatase TiO_2_ Nanoparticles with Exposed {001} Facets for Efficient Dye-Sensitized Solar Cells

**DOI:** 10.1038/srep12143

**Published:** 2015-07-20

**Authors:** Liang Chu, Zhengfei Qin, Jianping Yang, Xing’ao Li

**Affiliations:** 1 School of Science, Nanjing University of Posts and Telecommunications (NUPT), Nanjing 210046, P. R. China; 2 Key Laboratory for Organic Electronics & Information Displays (KLOEID) & Institute of Advanced Materials (IAM), Jiangsu National Synergistic Innovation Center for Advanced Materials (SICAM), School of Materials Science and Engineering (SMSE), Nanjing University of Posts and Telecommunications (NUPT), Nanjing 210046, P. R. China

## Abstract

Anatase TiO_2_ nanoparticles with exposed {001} facets were synthesized from Ti powder *via* a sequential hydrothermal reaction process. At the first-step hydrothermal reaction, H-titanate nanowires were obtained in NaOH solution with Ti powder, and at second-step hydrothermal reaction, anatase TiO_2_ nanoparticles with exposed {001} facets were formed in NH_4_F solution. If the second-step hydrothermal reaction was carried out in pure water, the H-titanate nanowires were decomposed into random shape anatase-TiO_2_ nanostructures, as well as few impurity of H_2_Ti_8_O_17_ phase and rutile TiO_2_ phase. Then, the as-prepared TiO_2_ nanostructures synthesized in NH_4_F solution and pure water were applied to the photoanodes of dye-sensitized solar cells (DSSCs), which exhibited power conversion efficiency (PCE) of 7.06% (*V*_OC_ of 0.756 V, *J*_SC_ of 14.80 mA/cm^2^, FF of 0.631) and 3.47% (*V*_OC_ of 0.764 V, *J*_SC_ of 6.86 mA/cm^2^, FF of 0.662), respectively. The outstanding performance of DSSCs based on anatase TiO_2_ nanoparticles with exposed {001} facets was attributed to the high activity and large special surface area for excellent capacity of dye adsorption.

Dye-sensitized solar cells (DSSCs), since the first report by Grätzel in 1991, have captured a lot of attentions due to the advantages of high power conversion efficiency (PCE), low cost, friendly to the environment, and simple fabrication process^1^,^2^,^3^. Traditionally, standard DSSC structure is the combination of photoanodes, dye sensitizers, redox electrolytes, and counter electrodes^4^,^5^,^6^. There, the photoanodes strongly affect the performance of DSSCs, which serve as scaffolds for dye molecules and the transport media for photo-generated electrons^7^,^8^,^9^,^10^. As a result, considerable efforts have been devoted to pursuing a more effective photoanode.

Titanium dioxide (TiO_2_) is the shared material for photoanode in DSSCs, because of the high chemical and optical stability, low toxicity, and appropriate band structure^11^,^12^,^13^. In DSSCs, the performance profoundly depends upon the morphology, crystalline phase, structure and exposed crystal facet of TiO_2_^14^,^15^,^16^. The previous studies indicated that anatase TiO_2_ single crystal with exposed {001} facets has good potency for dye adsorption and charge transfer^17^. Both theoretical and experimental studies showed that {001} facets of anatase TiO_2_ single crystal are extraordinarily reactive^18^, and the surface energy is 0.90 J/m^2^, which is much larger than 0.44 J/m^2^ surface energy of the usual {101} facets^19^. To date, there have been a large number of reports for preparing anatase TiO_2_ single crystal with appropriately exposed {001} facets for application of enhanced DSSCs, such as TiO_2_ nanotube^20^, anatase TiO_2_ nanosheets^21^, yolk@shell anatase TiO_2_ hierarchical microspheres^22^, and mesoporous TiO_2_ single crystals^9^. Yet it was reported that the ratio of {001} and {101} facet has impact on the performance of nanodevice because of the “surface heterojunction” of {001} and {101} surfaces, where appropriate but not great proportion of {001} facets is beneficial to the transfer and separation of photogenerated electrons and holes^23^,^24^.

Nowadays, the efficiency of DSSCs has been achieved 13% through the molecular engineering of porphyrin sensitizers^25^. It’s worth noting that the photoanode is TiO_2_ nanoparticle film because of the large specific surface area for loading dye molecules. To the best of our knowledge, it is a challenge to synthesize TiO_2_ nanoparticles with appropriately exposed {001} facets, which is the desired material for photoanodes of DSSCs. In this work, anatase TiO_2_ nanoparticles with 34% exposed {001} facets were synthesized *via* a two-step hydrothermal reaction method from Ti powder, which were further developed as efficient photoanodes for DSSCs. The first-step hydrothermal reaction of Ti powder in NaOH solution led to the formation of H-titanate nanowires after washing with HCl solution^26^, and the second-step hydrothermal reaction resulted in the formation of anatase TiO_2_ nanoparticles with exposed {001} facets in NH_4_F solution or random shape TiO_2_ nanostuctures with tiny impurity phase in pure water. Subsequently, the obtained TiO_2_ nanostructures were utilized as photoanodes of DSSCs, yielding PCE of 7.06% (*V*_OC_ of 0.756 V, *J*_SC_ of 14.80 mA/cm^2^, FF of 0.631) and 3.47% (*V*_OC_ of 0.764 V, *J*_SC_ of 6.86 mA/cm^2^, FF of 0.662), respectively. The result indicated the anatase TiO_2_ nanoparticles with 34% exposed {001} facets possess the characteristics of high activity and large special surface area for the excellent capacity to load dye molecules.

## Results

### Structure of anatase TiO_2_ nanoparticles

**Figure 1:**
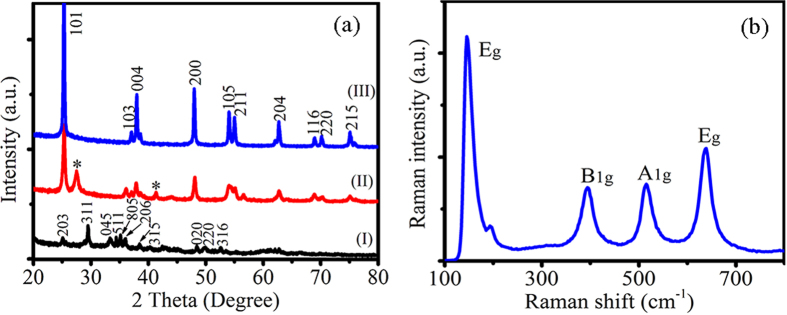
XRD powder patterns and Raman spectroscopy. (**a**) H-titanate nanowires corresponded well to H_2_Ti_5_O_11_·H_2_O phase (I), the obtained sample after the second-step hydrothermal reaction in pure water was indexed as mainly anatase TiO_2_ phase and few H_2_Ti_8_O_17_ phase and rutile TiO_2_ phase (II), the obtained sample in NH_4_F solution was indexed as pure anatase TiO_2_ phase (III). (**b**) Raman spectroscopy was taken to calculate the percentage of {001} facets as 34% by the peak intensity ratio of the E_g_ (at 144 cm^−1^) and A_1g_ (at 514 cm^−1^) peaks.

Figure 1(a) shows the powder XRD patterns of the as-grown H-titanate nanowires (I), as well as the obtained TiO_2_ nanostuctures synthesized in pure water (II) and in NH_4_F solution (III). In curve (I), there are several broadened diffraction peaks, which correspond well to the H_2_Ti_5_O_11_·H_2_O phase (PCPDFWIN, 44-0130). Thus, the Ti powder was reacted with NaOH solution, after washing with dilute HCl solution, the obtained sample was H_2_Ti_5_O_11_·H_2_O. Taking the H_2_Ti_5_O_11_·H_2_O nanowires as precursor in the following hydrothermal treatment in pure water, the obtained powder was mainly indexed as anatase TiO_2_ phase, as revealed in curve (II). Meanwhile, the another peaks besides anatase TiO_2_ phase indicate that the H_2_Ti_5_O_11_·H_2_O nanowires were not completely decomposed into pure anatase TiO_2_ phase. The peaks located at 27.71°and 41.86° were corresponded to (012) and (860) planes of H_2_Ti_8_O_17_ phase, respectively (PCPDFWIN, 36-0656). While the peaks located at 36.13° and 56.55° were assigned to (101) and (220) planes of rutile TiO_2_ phase (PCPDFWIN, 87-0710). More importantly, if the further hydrothermal reaction was taken in NH_4_F solution, a pure phase of anatase TiO_2_ (PCPDFWIN, 84-1286) was observed as the curve (III). Using the Scherrer equation, the average crystal size of anatase TiO_2_ nanostructures obtained in NH_4_F solution was about 45 nm estimated from the full width at half maximum of the (101) peak. Since no other diffraction peaks belonging to impurities are observed, suggesting that all the H_2_Ti_5_O_11_·H_2_O nanowires were completely converted to anatase TiO_2_ phase in NH_4_F solution. The diffraction peaks of curve (III) are sharper than that of curve (II), indicating the better crystallization and larger crystallites due to the enhanced effect of F^−^25. The obvious (004) diffraction peak of curve (III) suggests dominant crystal growth along the [001] direction^26^, which is typical for anatase TiO_2_ nanoparticles with exposed {001} facets. In order to quantitatively analyze the percentage of {001} facets, Raman spectroscopy was carried out as shown in Fig. 1(b)^24^,^27^. The peaks at 144, 394, 514, and 636 cm^−1^ suggest the typical anatase TiO_2_ phase, being consistent with the XRD results. The percentage of {001} facets was calculated as 34% by measuring the peak intensity ratio of the E_g_ (at 144 cm^−1^) and A_1g_ (at 514 cm^−1^) peaks^27^.

**Figure 2:**
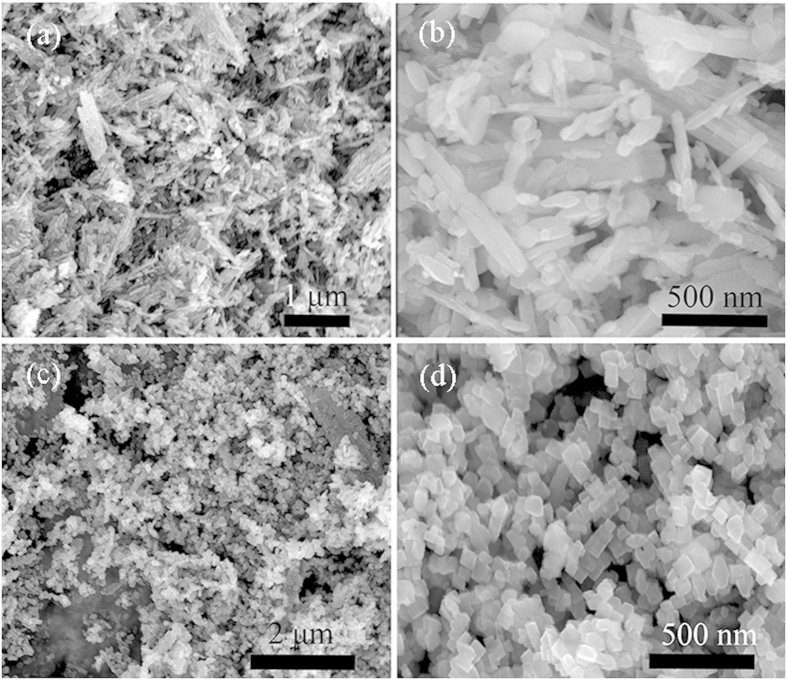
SEM images of the as-prepared TiO_2_. (**a**,**b**) Without NH_4_F, the obtained TiO_2_ nanostructures were random shapes. (**c**,**d**) With NH_4_F, regular TiO_2_ nanoparticles were obtained.

Figure 2 shows typical scanning electron microscopy (SEM) images of the TiO_2_ synthesized in pure water and in NH_4_F solution. After hydrothermal reaction and washing with HCl solution, the morphology of the as-prepared H_2_Ti_5_O_11_·H_2_O was nanowire structure (Supplementary Information Fig. 1S). After the further hydrothermal treatment at 200 °C for 48 h, the morphology of the H_2_Ti_5_O_11_·H_2_O nanowires undergone significant change. The H_2_Ti_5_O_11_·H_2_O nanowires were decomposed into random shape TiO_2_ nanostructures in pure water, as shown in Fig. 2 (a,b). There was complex morphology of nanorod, nanosphere, nanoellipsoid, irregular nanostructures, *etc*. Interestingly, the morphology of the TiO_2_ obtained in NH_4_F solution was regular nanoparticles with size of ~50 nm as illustrated in Fig. 2(c,d), in good agreement with the XRD measurement. The NH_4_F as morphology controlling agent led the H_2_Ti_5_O_11_·H_2_O nanowires to completely decomposing into regular TiO_2_ nanopaticles^27^.

**Figure 3:**
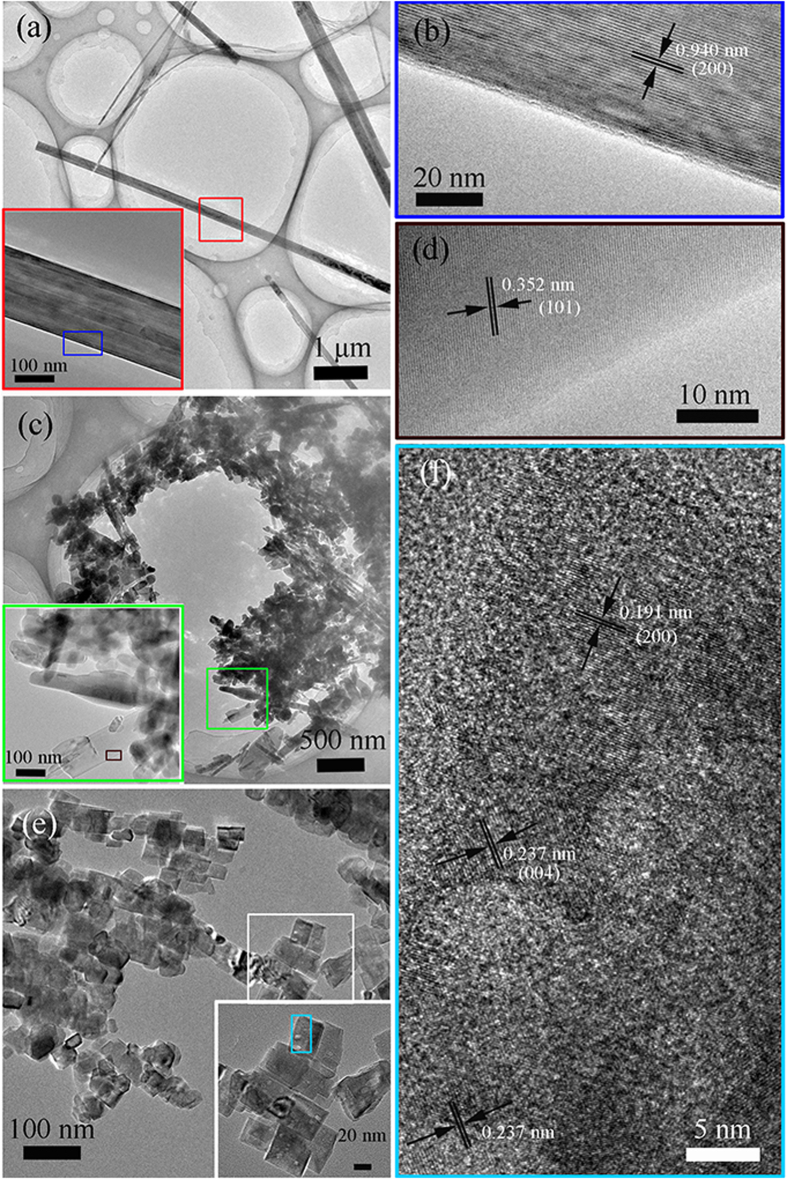
TEM and HRTEM images of the as-prepared samples. (**a**,**b**) H-titanate nanowires, (c, d) random shape TiO_2_ nanostructures obtained in pure water, (**e**,**f**) truncated octahedron TiO_2_ nanoparticles obtained in NH_4_F solution.

The transmission electron microscopy (TEM) was used to further characterize the crystal structure and morphology of H_2_Ti_5_O_11_·H_2_O and TiO_2_ nanostrctures in Figure 3. Fig. 3(a,b) shows the H_2_Ti_5_O_11_·H_2_O nanowire sample. The lattice fringes with distances of 0.940 nm in the HRTEM image of Fig. 3(b) corresponded well with (200) plane of H_2_Ti_5_O_11_·H_2_O phase. Figure 3(c) shows TEM image of random shape TiO_2_ nanostructures by the further hydrothermal reaction in pure water. The HRTEM image (Fig. 3(d)) corresponding the dark red-box area shows interplanar spacing of 0.352 nm, which matches well with (101) plane of anatase TiO_2_. When the second-step hydrothemal reaction was taken in NH_4_F solution, the H_2_Ti_5_O_11_·H_2_O nanowires were decomposed into regular TiO_2_ nanopaticles (Fig. 3(e)). The HRTEM image corresponding the cyan-box area in Fig. 3(e) was revealed in Fig. 3(f), where the interplanar spacing of 0.192 and 0.237 nm corresponded to (200) and (004) planes of anatase TiO_2_, respectively. In addition, the (004) plane indicated the anatase TiO_2_ single crystal with exposed {001} facets, and the shape of anatase TiO_2_ was truncated octahedron. Further, the {101} facets could be also observed through TEM technique (Supplementary Information Fig. 2S).

### Growth mechanism

**Figure 4:**
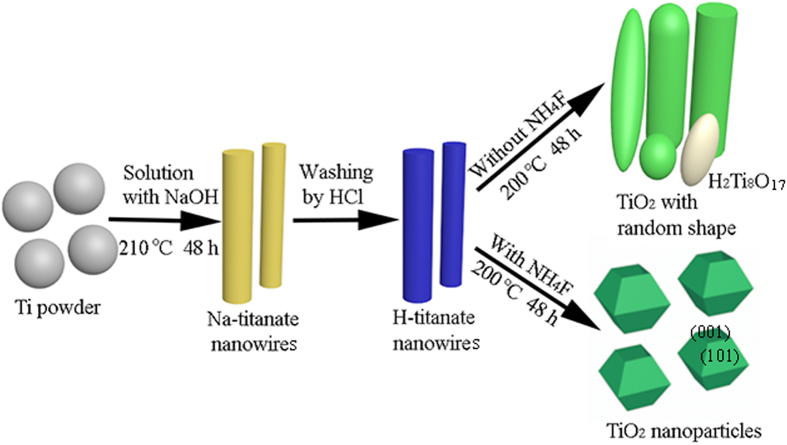
Schematic illustration of the synthetic route of TiO_2_. In pure water, random shape anatase TiO_2_ with few H_2_Ti_8_O_17_ and rutile TiO_2_ nanostrutures were formed. While in the present of NH_4_F, truncated octahedron TiO_2_ nanoparticles with exposed {001} facets were obtained.

Figure 4 illustrates the schematic of the preparation process of random shape TiO_2_ nanostrctures and truncated octahedron TiO_2_ nanoparticles with exprosed {001} facets *via* a two-step hydrothermal reaction process. At the first-step hydrothermal reaction, Ti powder reacted with NaOH to synthesize Na-titanate nanowires. Followed by washing with diluted HCl solution, the Na-titanate nanowires were transformed into H-titanate nanowires *via* cation exchange reaction. At the second-step hydrothermal reaction, the H-titanate nanowires precursors was under gone completely change in aqueous solution with or without NH_4_F. In this process, the H-titanate precursors experienced a dissolution and nucleation process during the hydrothermal treatment^28^. In pure water, the dissolution occurred without any restraint, thus TiO_2_ nanostructures with random shape distribution were obtained. Moreover, the nucleation was not thorough, and there was few H_2_Ti_8_O_17_ phase and TiO_2_ rutile phase. When the H-titanate precursors were performed dissolution and nucleation in NH_4_F solution, single crystal anatase TiO_2_ nanoparticles with exposed {001} facets were obtained. At the dissolution process, the existing of F^−^ ions could be bonded with Ti atom to reduce the surface energy of the {001} facets to lower than that of the {101} facets, resulting in exposing {001} facets during nucleation^29^,^30^. Besides, F^−^ ions acted as morphology controlling agent to control the shape of TiO_2_ nanostructures during nucleation, and the shape of TiO_2_ nanoparticles was truncated octahedron, as shown in Fig. 4.

### Characterization of Photovoltaic Performance

**Figure 5:**
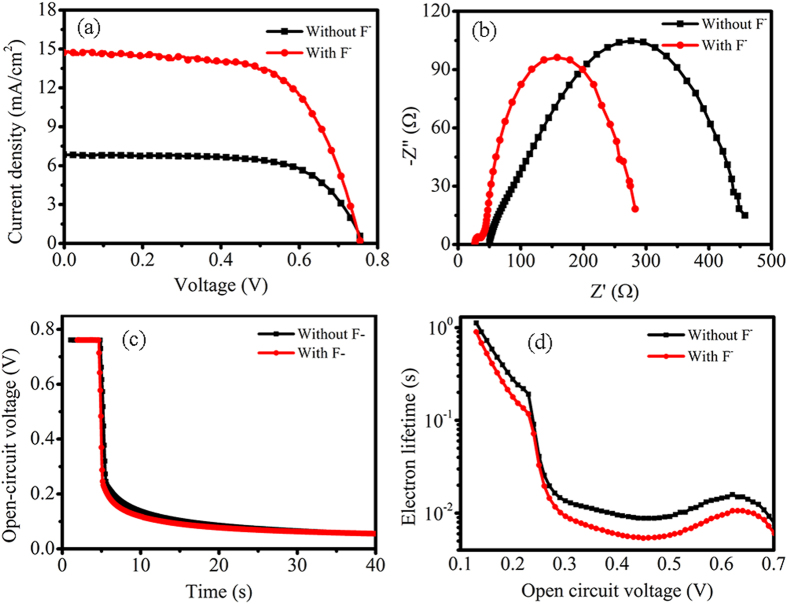
Photovoltaic characteristics of the DSSCs based on random shape TiO_2_ nanostructures (noted “Without F^−^”) and truncated octahedron TiO_2_ nanoparticles with exposed {001} facets (noted “With F^−^”). (**a**) I-V curves. (**b**) Nyquist plots by EIS measurements. (**c**) Open-voltage decay measurement upon turning off the illumination. (d) Electron lifetime determined from the data of (**c**).

The obtained TiO_2_ powders were mixed with some additive agents to make TiO_2_ pastes, and then the TiO_2_ pastes were coated on TiCl_4_-treated FTO glasses by doctor-blading method to realize photoanodes after annealing. The thickness of the TiO_2_ photoanodes was 13.5 μm (Supplementary Information Fig. 3S). Figure 5(a) indicates the current density-voltage (I-V) curves of the DSSCs based on random shape TiO_2_ nanostructures (noted “Without F^−^”) and truncated octahedron TiO_2_ nanoparticles with 34% exposed {001} facets (noted “With F^−^”). Table 1 listed the corresponding detailed photovoltaic parameters, including the open-circuit voltage (*V*_OC_), short-circuit current density (*J*_SC_), fill factor (FF), and PEC. The larger PCE 7.06% of DSSCs (noted “With F^−^”) was mainly rooted in the *J*_SC_, which increased from 6.86 to 14.80 mA/cm^2^. Generally, *J*_SC_ can be approximated as following expression^31^:


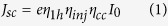


where *e* is the elementary charge, *η*_l*h*_ is the light-harvesting efficiency related to the amount of adsorbed dye molecules and the light-scattering properties, *η*_*inj*_ is the charge-injection efficiency, *η*_*cc*_ is the charge-collection efficiency relied on competition between charge recombination and collection, and *I*_*0*_ is the light flux. Here, *η*_*inj*_ is suggested to be of the same value, because of the injection both from the TiO_2_ material to N719 dye.

Electrochemical impedance spectroscopic (EIS) measurements were conducted in the dark under a bias of 0.75 V to evaluate the charge transfer and recombination as the Nyquist plots in Fig. 5(b)^32^. The radius of semicircle in Nyquist plots revealed the charge-transfer resistance (*R*_ct_) between TiO_2_/dye/electrolyte interfaces. The slightly larger one based on the random shape TiO_2_ nanostructures (Without F^−^) indicated a slow charge recombination at the TiO_2_/dye/electrolyte interfaces. Namely, the electron lifetime in photoanodes based on the random shape TiO_2_ nanostructures was slightly longer. Moreover, the electron lifetime (*τ*) was calculated using the following equation^33^,^34^:


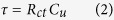


where *C*_*u*_ is the corresponding chemical capacitance. The corresponding values of *R*_ct_ (243.3 and 237.1 Ω) and *C*_*u*_ (7.5534 × 10^−4^ and 7.1165 × 10^−4^ F) can be obtained by simulation using the Zview software. The electron lifetime was 0.184 (Without NH_4_F) and 0.179 s (With F^−^) of DSSCs, respectively. Meanwhile, the open-voltage decay method was employed to further investigate the electron lifetime of the two cells, as shown in Fig. 5(c). From the open-voltage decay rate, the electron lifetime (*τ*) can be calculated by the following equation^35^,^36^:


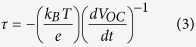


where *k*_*B*_ is the Boltzmann constant, and *T* is room temperature. The calculated data of *τ* were plotted in Fig. 5(d). It was observed that the electron lifetime based on random shape TiO_2_ nanostructures was slightly longer than that based on TiO_2_ nanoparticles. The possible reason was that there were some nanorods and relatively large nanoparticles in random shape TiO_2_ nanostructures, which were in favour of charge transfer. Therefore, it dos no outstanding difference to the charge transfer, and the value of *η*_*cc*_ of DSSCs (noted “Without NH_4_F”) was little larger than that one (noted “With NH_4_F”). In other words, the increasing of *J*_SC_ at over twice was not from the factor of *η*_*cc*_.

**Figure 6:**
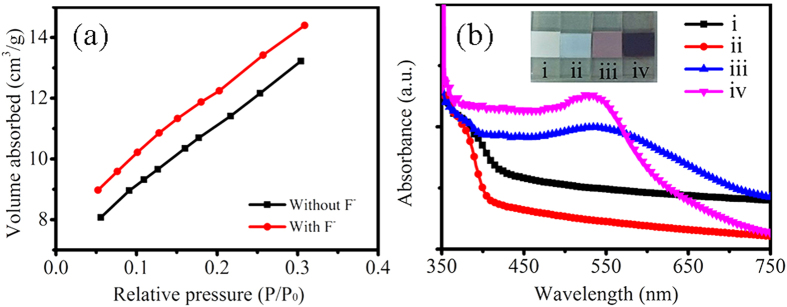
N_2_ adsorption isotherms and absorption spectra. (**a**) N_2_ adsorption isotherms of random shape TiO_2_ nanostructures (noted “Without F^−^”) and truncated octahedron TiO_2_ nanoparticles (noted “With F^−^”). (**b**) UV-Vis absorption spectrum of random shape TiO_2_ nanostructure films (without/with sensitizing, i/iii) and truncated octahedron TiO_2_ nanoparticle films (without/with sensitizing, ii/iv) on FTO substrates. The inset shows optical images of TiO_2_ films (without/with sensitizing) on FTO substrates.

It is well-known that the nanoparticle films have large special surface area to load more dye molecules^1^. Therefore, the special surface area was checked by Brunauer-Emmett-Teller (BET) data as shown in Fig. 6(a). The BET surface area was measured as 40.9 and 44.6 m^2^/g for random shape TiO_2_ nanostructures and TiO_2_ nanoparticles, respectively. The special surface area of TiO_2_ nanoparticles was slightly bigger than that of the random shape TiO_2_ nanostructures. More importantly, exposing highly reactive {001} facets of TiO_2_ can enhance dye adsorption^17^. Thus, we investigated the amount of absorbed dye molecules to elucidate the factor of *η*_l*h*_. The optical image of TiO_2_ and sensitized-TiO_2_ films on FTO substrates was shown as the inset in Fig. 6(b). The color of random shape TiO_2_ nanostructure films (i) was lutescent, while the color of TiO_2_ nanoparticle films (ii) was pure white. After being sensitized by N719 dye, the color of the sensitized-TiO_2_ films based on TiO_2_ nanoparticles (iv) was darker red than that of based on random shape TiO_2_ nanostructures (iii), indicating that the TiO_2_ nanoparticles with exposed {001} facets absorbed more dye molecules. The UV-vis absorbance measurements in Fig. 6(b) revealed the sensitized-TiO_2_ films based on TiO_2_ nanoparticles (iv) had a stronger visible absorption, because of more amount of loading dye molecules. The absorbed dye amounts were calculated from UV-vis absorbance measurements of the concentration desorbed N719 dye in NaOH solution by using Lambert-Beer’s Law^37^,^38^. The absorbed dye amount of photoanodes based on TiO_2_ nanopartiles with exposed {001} facets was about four times than that of based on random shape TiO_2_ nanostructures, as listed in Table 1. The TiO_2_ nanoparticles with exposed {001} facets had excellent capacity for adsorption of dye molecules. As a consequence, anatase TiO_2_ nanoparticles with exposed {001} facets were efficient photoanodes for DSSCs.

## Discussion

Anatase TiO_2_ nanoparticles with 34% exposed {001} facets have been successfully synthesized from Ti powder *via* two-step hydrothermal reaction process. The first -step hydrothermal reaction was alkaline hydrothermal reaction to obtain H-titanate nanowires. At the second-step hydrothermal reaction, the H-titanate nanowires were decomposed into random shape anatase TiO_2_ nanostrctures with few impurity in pure water or truncated octahedron anatase TiO_2_ nanopaticles with 34% exposed {001} facets in NH_4_F solution. The DSSCs based on anatase TiO_2_ nanopaticles with 34% exposed {001} facets showed outstanding performance of efficiency 7.03%, which was about twice than that of based on random shape TiO_2_ nanostructures. The high performance was ascribed to that the anatase TiO_2_ nanopaticles with 34% exposed {001} facets own high activity and large special surface area for excellent capacity of absorbing dye molecules. We anticipate that the anatase TiO_2_ nanopaticles with 34% exposed {001} facets open up a promising avenue for efficient TiO_2_-based photoelectric nanodevices.

## Methods

### Materials

Fuorinated tin oxide (FTO, ~7 Ω/cm^2^) glasses were bought from Nippin Sheet Glass Co., Ltd. Sodium hydroxide (NaOH, 96.0%), titanium tetrachloride (TiCl_4_, 99.0%), ammonium fluoride (NH_4_F, 96.0%), hydrochliric acid (HCl, 36%~38%wt), ethanol (99.7%) and acetone (99.5%) were purchased from Sinopharm Chemical Reagent Co. Ltd. Ruthenium 535-bisTBA (N719) was purchased from Solaronix. Guanidinium thiocyanate (GuSCN, 99.0%) was from Amresco. Titanium powder (Ti, 99.99%), lithium iodide (LiI, 99.999%), iodine (I_2_, 99.99%), 1-methyl-3- propylimidazolium iodide (PMII, 98%), 4-tert-butylpyridine (4-TBP, 96%) and tert-butyl alcohol (99.5%) were obtained from Aladdin. Acetonitrile (99.8%) and valeronitrile (99%) were from Alfa Aesar. All solvents and chemicals were reagent grade and were used without further purification.

### Synthesis of H-titanate nanowires

Na-titanate nanowires were firstly synthesized by alkali hydrothermal reaction of Ti powder in NaOH solution. First, 70 mL of 10 M NaOH solution was obtained under magnetic stirring. Then 0.2 g Ti powder was added into the above NaOH solution and stirred for 10 minimums again. The final solution was transferred to a 100 ml Teflon-lined stainless steel autoclave and loaded into an oven. The temperature was set 210 °C for 48 hours and then cooled down to room temperature naturally. After the hydrothermal treatment, the obtained Na-titanate nanowires were completely washed with 0.1 M HCl solution to replace Na^+^ with H^+^. Subsequently, the H-titanate nanowires were washed with deionized water several times.

### Synthesis of anatase TiO_2_ nanopaticles with exposed {001} facets

The above total H-titanate nanowires were added into 100 mL Teflon-lined stainless steel autoclave containing 70 ml deionized water with or without adding 0.25 M NH_4_F. Afterward, the autoclave was loaded into an oven at 200 °C for 48 hours and then cooled down to room temperature naturally. After the hydrothermal reaction, the obtained TiO_2_ powders were collected from the solution, and washed with deionized water and ethanol for several times by centrifugation. Finally, the powders were dried at 80 °C over night. The obtained dry powder was anatase TiO_2_ nanopaticles with exposed {001} facets in NH_4_F solution or random shape TiO_2_ nanostuctures with few impurity in pure water.

### Preparation of TiO_2_ photoanode

1 g TiO_2_ powder was mixed evenly under magnetic stirring in a mixture of 0.2 mL acetic acid, 3.0 g terpineol, 0.5 g ethyl cellulose and some ethanol to form a slurry, the slurry was milled in a mortar for about 20 min, and then dispersed with ultrasonic for 10 min to prepare viscous white TiO_2_ paste. The FTO glasses were washed with detergent and sonicated in deionized water, acetone and ethanol for 20 min, respectively. After dried under flowing argon gas, the cleaned FTO glasses were soaked into 0.04 M TiCl_4_ solution at 70 °C for 30 min to form a compact TiO_2_ layer, and then rinsed with deionized water and ethanol. The TiO_2_ pastes were printed onto the TiCl_4_-treated FTO glasses by doctor-blading method. Then the printed TiO_2_ layers were annealed at 125 °C for 15 min, at 325 °C for 5 min, at 375 °C for 5 min, at 450 °C for 15 min, and then at 500 °C for 15 min in a muffle furnace. The annealed TiO_2_ layers were immersed into 40 mM TiCl_4_ solution at 70 °C for 30 min again, and after being rinsed with deionized water and ethanol, the films were sintered at 500 °C for 30 min in muffle furnace. After the temperature was cooled to about 80 °C, the TiO_2_ pohtoanodes were immersed into 0.5 mM N719 dye in acetonitrile/tert-butanol (V:V/1:1), and kept for 16 h at room temperature. The sensitized TiO_2_ photoanodes were washed with acetonitrile to remove the possible physically-adsorbed dye molecules.

### Fabrication of DSSCs

The Pt counter electrodes were deposited by magnetron sputtering on cleaned FTO glasses. Sputtering was performed using a Pt (99.99% purity) target in an Ar ambient atmosphere at 100 W. For fabricating DSSCs, the Pt counter electrodes were buckled on the sensitized-TiO_2_ photoanodes, which were sealed using a 50 μm plastic sheet and the internal space was filled with a liquid electrolyte. The electrolyte was composed of 0.6 M PMII, 0.05 M LiI, 0.03 M I_2_, 0.1 M GuSCN and 0.5 M 4-TBP in acetonitrile and valeronitrile (V:V/85:15). The active area of the solar cell was 0.15 cm^2^ without a mask.

### Measurement

The crystal structure and phase purity of the obtained powders were investigated using a powder X-ray diffractometer (XRD, PANalytical B.V., The Netherlands) with Cu-Kα (λ = 0.15418 nm) radiation. Raman measurement was carried out using a Raman spectroscopy (LABRAM HR800, France) with a 514.5 nm argon ion laser of 200 μm spot size for excitation. The size and morphology of the samples were recorded by field emission scanning electron microscopy (SEM, FEI NOVA NanoSEM 450). Transmission electron microscopy (TEM) and and high-resolution TEM (HRTEM) images were performed by TEM (FEI Tecnai G^2^ 20 UTwin) or aberration-corrected TEM (FEI Titan G^2^ 60-300). The sample was prepared by drop casting ethanolic dispersion of tiny TiO_2_ powder onto a carbon coated Cu grid. The Brunauer-Emmett-Teller (BET, V-Sorb 2800P) was carried out to measure the surface area of the samples. The current density-voltage (*I-V*) measurements, open-voltage decay measurements, and electrochemical impedance spectroscopy (EIS) measurements were performed by an Autolab electrochemic workstation (modelAUT84315, The Netherlands). UV-Vis absorption spectrometry (UV-2550, Shimadzu) was employed to test the absorption spectra. The illumination intensity was AM 1.5G (100 mW/cm^2^, calibrated with a Si photodiode) using a solar simulator (Newport, USA). The electrochemical impedance spectroscopy (EIS) measurements were scanned in dark condition at a bias of 0.75 V with an amplitude of 10 mV in a frequency range from 100 kHz to 0.1 Hz. For testing the adsorbed dye amount of the TiO_2_ working eletrodes, the sensitized-TiO_2_ samples desorbed the dye into 0.1 M NaOH solution. The measured absorption spectra were used to calculate the amount of the adsorbed dye amount, expressed in terms of moles of dye anchored per projected unit area of the photoanodes.

## Additional Information

**How to cite this article**: Chu, L. *et al.* Anatase TiO_2_ Nanoparticles with Exposed {001} Facets for Efficient Dye-Sensitized Solar Cells. *Sci. Rep.* **5**, 12143; doi: 10.1038/srep12143 (2015).

## Supplementary Material

Supplementary Information

## Figures and Tables

**Table 1:** Photovoltaic parameters of the DDSCs based on random shape TiO_2_ nanostructures (noted “Without F^−^”) and truncated octahedron TiO_2_ nanoparticles with 34% exposed {001} facets (noted “With F^−^”).

**Samples**	***J*_*sc*_(mA/cm^2^)**	***V*_oc_(V)**	**FF**	**PCE**	**Adsorbed dye (nmol/cm^2^)**^a^
Without F^−^	6.86	0.764	0.662	3.47%	32.6
With F^−^	14.80	0.756	0.631	7.06%	122.9

^a^Dye-adsorbed films with a dimension of 0.9 cm^2^ were used for estimating the adsorbed dye concentration.
