# Supplementary material for: Anatase TiO2 Nanoparticles with Exposed {001} Facets for Efficient Dye-Sensitized Solar Cells
Source: Sci Rep. 2015 Jul 20;5:12143. doi: 10.1038/srep12143 (PMC4507182; doi:10.1038/srep12143)
Supplement: Supplementary Information [file srep12143-s1.doc]

Supplementary Information for

Anatase TiO2 Nanoparticles with Exposed {001} Facets for Efficient Dye-Sensitized Solar Cells

Liang Chu^1^, Jianping Yang^1^* & Xing’ao Li^2^*

^1^ School of Science, Nanjing University of Posts and Telecommunications (NUPT), Nanjing 210046, P. R. China

^2^ Key Laboratory for Organic Electronics & Information Displays (KLOEID), Institute of Advanced Materials (IAM), School of Materials Science and Engineering (SMSE), Nanjing University of Posts and Telecommunications (NUPT), Nanjing 210046, P. R. China

**Correspondence and requests for materials should be addressed to J.P.Y.** ([yangjp@njupt.edu.cn](mailto:yangjp@njupt.edu.cn)) **X.A.L.**  **and** ([iamxali@njupt.edu.cn](mailto:iamxali@njupt.edu.cn))


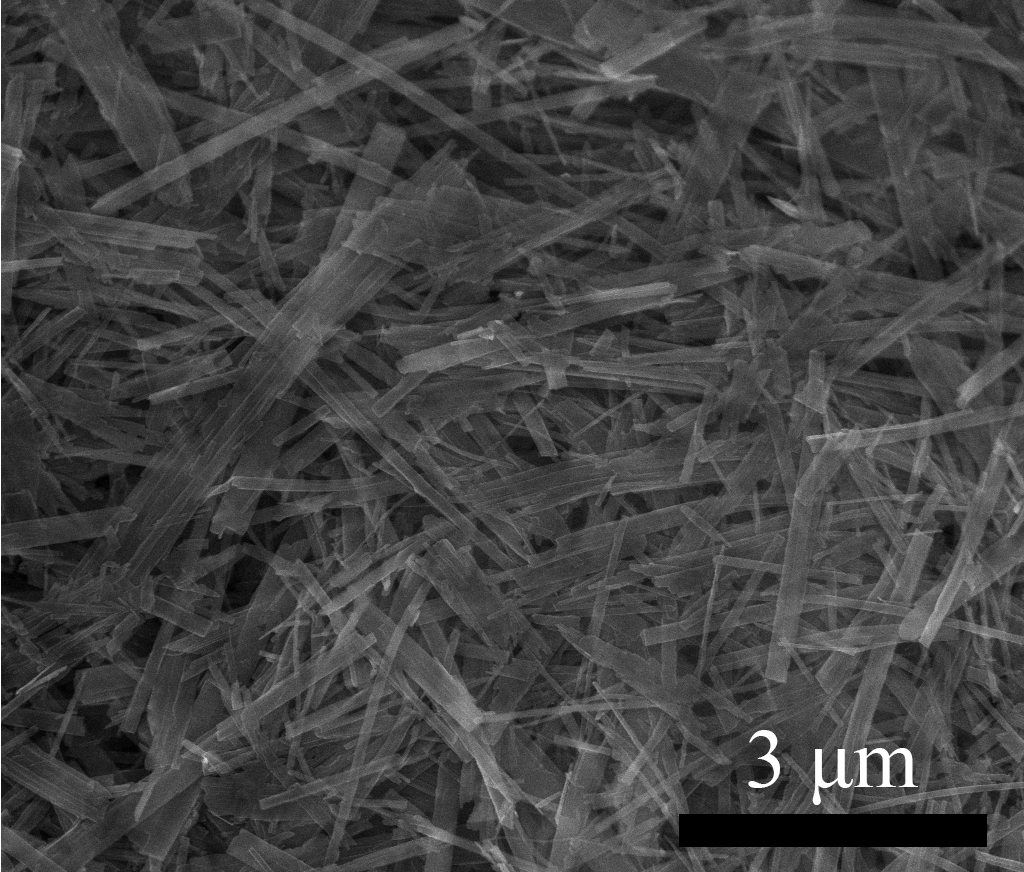


Figure S1 The SEM image of the obtained H-titanate nanowires. The morphology is nanowire structure.


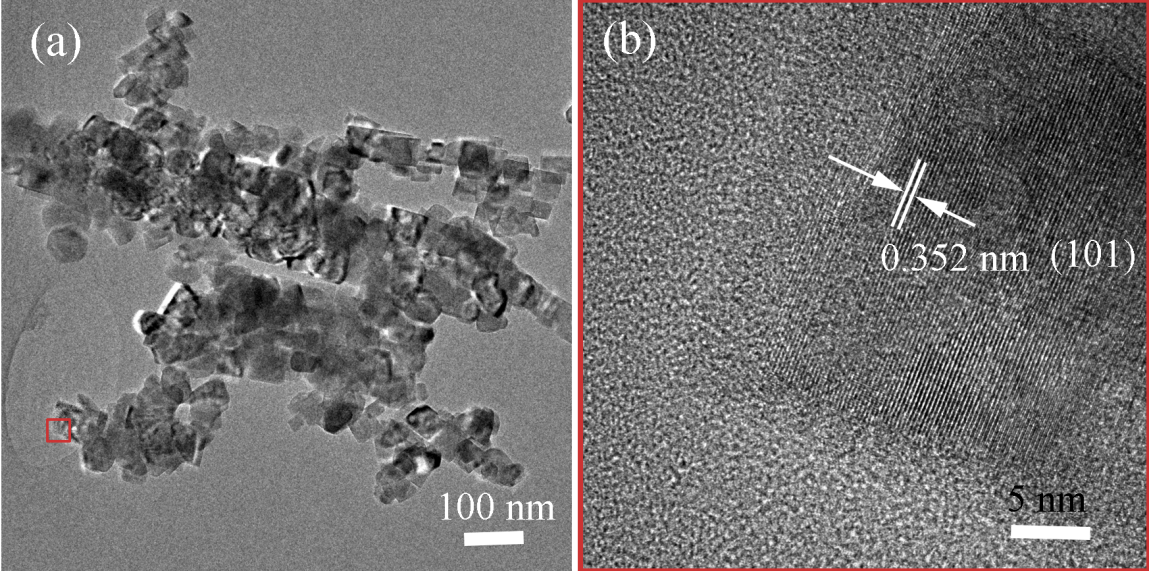


Figure S2｜ (a) The TEM image of TiO_2_ nanoparticles, (b) the HRTEM image corresponding the red-box area in (a). The lattice fringes of 0.352 nm is well agreement with the (101) face of anatase TiO_2_ [single](../../../../C:/Users/Administrator/AppData/Local/Yodao/DeskDict/frame/20141228184717/javascript:void(0)%3B) [crystal](../../../../C:/Users/Administrator/AppData/Local/Yodao/DeskDict/frame/20141228184717/javascript:void(0)%3B).


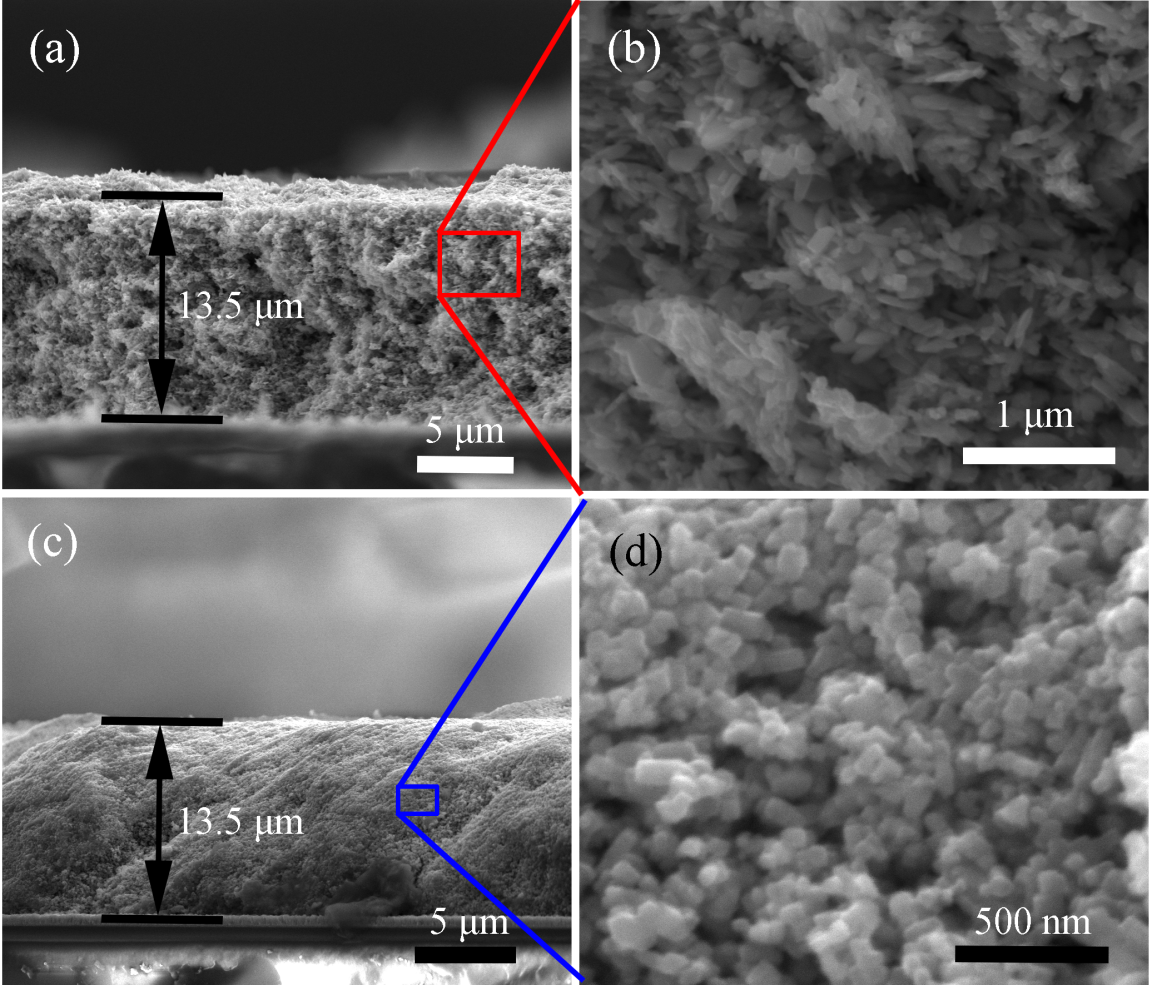


Figure S3｜The cross-section SEM images. (a) random shape TiO_2_-based photoanode, (b) the enlarged image corresponding the red-box in (a); (c) TiO_2_ nanopartice-based photoanode, (d) the enlarged image corresponding the blue box in (c). The above two photoanodes have the same thickness of 13.5 μm. From the enlarged images, the [interspace](../../../../C:/Users/Administrator/AppData/Local/Yodao/DeskDict/frame/20141228184717/javascript:void(0)%3B) between [interparticles](../../../../C:/Users/Administrator/AppData/Local/Yodao/DeskDict/frame/20141228184717/javascript:void(0)%3B) in photoanodes is well-distributed.
